# Indian plate paleogeography, subduction and horizontal underthrusting below Tibet: paradoxes, controversies and opportunities

**DOI:** 10.1093/nsr/nwac074

**Published:** 2022-04-21

**Authors:** Douwe J J van Hinsbergen

**Affiliations:** Department of Earth Sciences, Utrecht University, Utrecht 3584 CB, Netherlands

**Keywords:** collision, orogenesis, subduction, reconstruction, Himalaya, Tibet

## Abstract

The India–Asia collision zone is the archetype to calibrate geological responses to continent–continent collision, but hosts a paradox: there is no orogen-wide geological record of oceanic subduction after initial collision around 60–55 Ma, yet thousands of kilometers of post-collisional subduction occurred before the arrival of unsubductable continental lithosphere that currently horizontally underlies Tibet. Kinematically restoring incipient horizontal underthrusting accurately predicts geologically estimated diachronous slab break-off, unlocking the Miocene of Himalaya–Tibet as a natural laboratory for unsubductable lithosphere convergence. Additionally, three endmember paleogeographic scenarios exist with different predictions for the nature of post-collisional subducted lithosphere but each is defended and challenged based on similar data types. This paper attempts at breaking through this impasse by identifying how the three paleogeographic scenarios each challenge paradigms in geodynamics, orogenesis, magmatism or paleogeographic reconstruction and identify opportunities for methodological advances in paleomagnetism, sediment provenance analysis, and seismology to conclusively constrain Greater Indian paleogeography.

## Teaser

Indian continental lithosphere horizontally underthrusts and uplifts Tibet, providing the archetype of continental collision. This paper shows how this situation formed in the last 25 million years and outlines the paleogeographic and geodynamic consequences of pre-Miocene models, and the opportunities these provide for fundamental earth scientific research.

## INTRODUCTION

With major continents being too buoyant to subduct—the reason why they can become billions of years old—colliding continents are associated with subduction arrest, plate reorganization, orogenesis [1], seaway closure, mountain building, and atmospheric barrier formation [2]. The orogen at the India–Asia continental collision zone is the archetype to calibrate the relationships between collision, orogenic architecture, history, and dynamics, resulting magmatism and mineralization, as well as climatic and biological responses [2–6]. But long-standing paradoxes and controversies in tectonic history have led to an impasse, making using the full potential of the archetype difficult.

Geophysical imaging has revealed that Indian continental lithosphere has horizontally underthrust the Tibetan upper plate [7–12]. This is consistent with the paradigm of unsubductability of thick continental lithosphere [1] and offers opportunities to study the dynamics of and response to convergence between buoyant lithospheres [13]. But Indian lithosphere only reaches ∼400–800 km north of the Himalayan front [7–12] and, according to kinematic reconstructions of Indian plate consumption [9,11,14] and geological estimates of the last slab break-off in the Himalaya [15], accounts for only the last 25–13 Ma (diachronous along-strike) of India–Asia convergence [9,14]. Paradoxically, the youngest unequivocal geological records of plate-boundary-wide oceanic subduction between India and Asia are older than 60 Ma [16–18], after which >4000 km of India–Asia plate convergence occurred [19,20]. So between the geologically recorded collision and the onset of horizontal underthrusting of Indian lithosphere, thousands of kilometers of post-collisional subduction occurred.

This paradox is not readily explained by dynamic models of continental collision. These rather portray a process of ∼10 Ma, during which a few hundred kilometers of one continental margin is dragged down below another causing deformation of both margins, after which convergence stops, the slab detaches and the deformed belt rebounds and uplifts [21]. Long-standing controversy in the geological debate on the India–Asia collision history comes from different solutions to explain this paradox. Endmember solutions fall into three classes that fundamentally differ in post-collisional paleogeography of the Indian plate. The first endmember predicts that all post-collisional subduction consumed continental lithosphere [18,22,23] and the second and third infer that after initial collision, oceanic lithosphere remained to the north [6,24–27], or to the south [9,28] of the initial collision zone, which subsequently subducted ‘post-collision’. The former option challenges the paradigm of wholesale continental unsubductability. While it has become clear that thinned continental lithosphere may become dense enough to subduct without leading to subduction arrest and slab break-off, e.g. due to eclogitization during burial, in numerical experiments [29] as well as in orogens elsewhere [30], the sedimentary upper crust is decoupled from subducted continental lithosphere and remains behind in orogenic belts. If all of Greater India was continental, far more continental crust is subducted than suggested by the upper-crustal remains found in the Himalaya; if true, this is key to advancing the understanding of geodynamics [23]. The latter options challenge paradigms of orogenic architecture and evolution ensuing from oceanic subduction [22,31] and, if true, hold key lessons for reconstructing paleogeography from orogenic archives [30]. In all cases, the records of magmatism, deformation and topographic rise in Tibet and the Himalaya between the onset of collision and the onset of horizontal underthrusting occurred in the context of, and contain key information on atypical subduction, either in terms of the nature of the downgoing plate or in terms of the orogenic and magmatic response.

In the last decade, the controversy on India's paleogeography has reached an impasse: each of the endmember scenarios is argued for and against based on the same types of data, notably sediment provenance constraining upper-plate sediments arriving on lower plate continental margins [6,9,18,32,33], paleomagnetic data constraining paleolatitudes of continental margins and arcs [26,28,34–37] and seismic tomographic images revealing locations of past subduction zones [11,14,38,39]. Even though the volume of these databases has rapidly increased in recent years, they have mostly focused on testing the kinematic and paleogeographic predictions of each endmember model without leading to a consensus. This paper rather aims to explore the unique opportunities that each of these endmembers holds for the archetype to challenge and develop paradigms of geodynamics, orogenesis, and environmental response.

This paper aims to (i) attempt at formulating the paradox and explaining the controversy and the key predictions of each proposed class of explanations; (ii) review geological constraints on Indian plate subduction provided by the Himalayan mountains that consist of offscraped upper-crustal rocks derived from Indian plate lithosphere and accreted to the upper plate, and on coeval upper-plate geological evolution of the Tibetan Plateau; (iii) use these constraints to identify which tectonic and magmatic reorganizations coincide with horizontal Indian underthrusting, and aim to identify the natural laboratory to analyse the dynamics of non-subductable lithosphere convergence; (iv) discuss ways forward to reconcile existing data sets and find novel ones to break through the impasse in Greater India paleogeography reconstruction and show the opportunities that each of the three endmember scenarios would provide in using the India–Asia archetype to constrain the geological and dynamic consequences of its atypical post-collisional subduction.

## REVIEW

### The paradox: underthrust versus subducted Indian plate lithosphere

**Figure 1.**
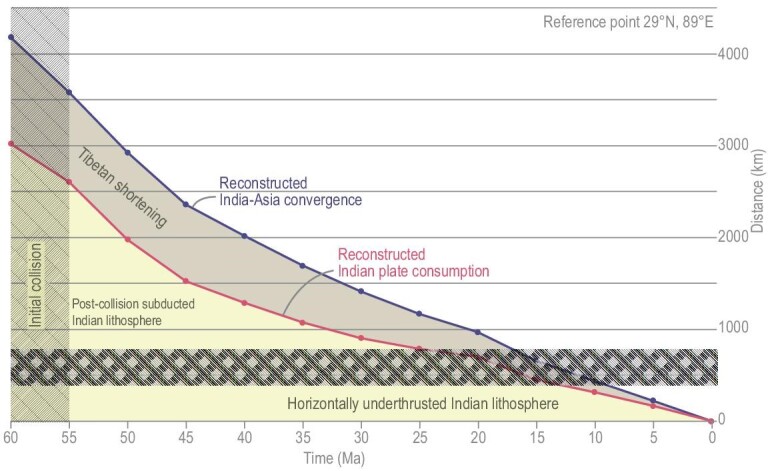
Reconstructed India–Asia convergence [20], which, when corrected for Tibetan shortening [9], predicts Indian plate subduction/underthrusting for the last 60 Ma. The amount of post-collisional subduction is a function of initial collision age recorded in the Himalaya (60–55 Ma) [17,18,43] and the width of horizontally underthrust India, which varies along-strike from 400 to 800 km (at the longitude of the reference location, this width is ∼400 km, Fig. 2).

A key question in the analysis of the India–Asia collision history and dynamics is where and how post-collisional convergence has been accommodated. Kinematic reconstructions have shown that ∼1000–1200 km of Cenozoic convergence was accommodated by shortening and extrusion in the overriding plate of Tibet [9,40,41]. Reconstructing this convergence in the mantle reference frame aligns the southern Eurasian margin with underlying slabs imaged by seismic tomography and in the paleomagnetic reference frame satisfies first-order vertical-axis rotations and south Tibetan paleolatitudes for the Cretaceous and Paleogene [9]. This reconstructed shortening of Tibet is by far the largest amount of intraplate shortening recorded in post-Paleozoic orogens [30]. Shortening records of the Indian-plate-derived thin-skinned Himalaya fold-thrust belt give somewhat smaller numbers, between 600 and 900 km [42]. It is puzzling that post-collisional convergence far exceeds these numbers: the earliest estimates for post-collisional convergence assumed a 45-Ma collision [40], which would generate a shortening deficit of ∼1000 km, but stratigraphic ages of the oldest foreland basin clastics in the northernmost continental rocks of the Himalaya as well as ages of (U)HP metamorphism in continent-derived rocks in the northern Himalaya have pushed the estimated initial collision age backward, to ∼60–55 Ma [16,17,43]. India–Asia plate circuits constrained by magnetic anomalies predict 3500 and 4500 km of post-60-Ma convergence at the longitude of the western and eastern Himalayan syntaxis, respectively [19,20] (Fig. 1). Much of the post-collisional subduction has thus not left an accreted rock record, either because of wholesale subduction or (subduction-) erosion of previously accreted records.

Seismological research in the last two decades has painted a detailed image of the mantle below India and Tibet that helps in identifying where lost lithosphere may now reside. First, lithosphere below Tibet is ≤260 km thick, which was at first surprising [44]: major lithospheric thickening associated with intraplate shortening is predicted to lead to convective instability of lithosphere, which will then delaminate [45]. However, since then, the thick lithosphere below Tibet has become interpreted as horizontally underthrust Indian crust and continental mantle lithosphere [7–12]. Tibetan lithosphere has indeed delimanated: Indian continental crust appears to directly underlie Tibetan crust and is not intervened by a thick lithospheric mantle [12]. In addition, seismic tomographic evidence for bodies of high-velocity material that may represent delaminated Tibetan lithosphere have been identified in the upper mantle below the horizontally underthrust Indian lithosphere, suggesting delamination prior to underthrusting [46]. Moreover, recent seismological analysis has shown that delamination is not restricted to Tibet, but also affected the Yunnan region to the southeast of the eastern Himalayan syntaxis, where a conspicuous, circular-shaped hole in the continental lithosphere is underlain by a body of high-velocity material at the base of the upper mantle [47].

**Figure 2.**
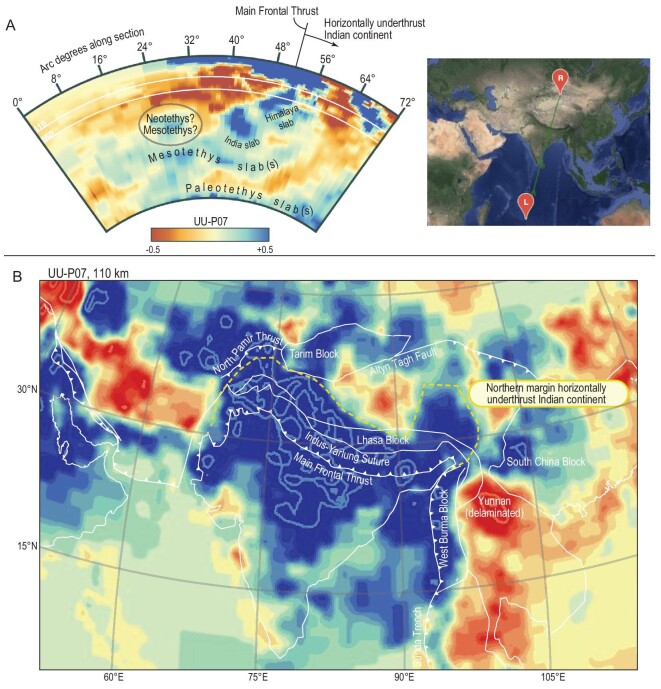
Seismic tomographic images taken from the UU-P07 tomography model [48,99]. (A) Vertical section from the Indian Ocean to Central Asia (drawn using the Hades Underworld Explorer, www.atlas-of-the-underworld.org). Deep, flat-lying slabs relate to Mesozoic Paleotethys and Mesotethys subduction during the amalgamation of Tibetan terranes [14]. The India slab contains the bulk of Neotethys lithosphere that subducted northward below the Lhasa terrane, whereas the northward subducted but overturned Himalaya slab contains subducted Greater Indian lithosphere [9,11,14,38,39]. Horizontally underthrust Indian continental lithosphere protrudes northward from the Main Frontal Thrust over a distance of 400–800 km, varying along-strike [7–10,14]. (B) Horizontal cross section at 110-km depth through the UU-P07 tomography model, overlain by outlines of modern geology and geography. The yellow dotted line depicts the outline of the northern margin of horizontally underthrust Indian continent below Tibet, protruding ∼800 km northward north of the Himalayan syntaxes, decreasing to ∼400 km towards ∼90°E [7,9,10].

**Figure 3.**
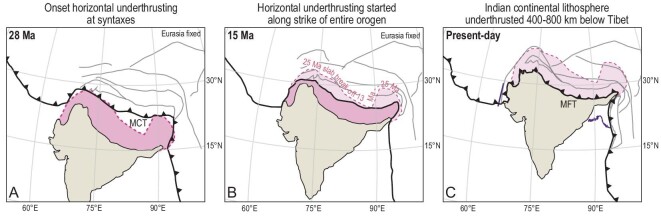
Reconstructions of the diachronous onset of horizontal Indian underthrusting at (A) 28 Ma, (B) 15 Ma and (C) the present day, using the outline of horizontally underthrust continental lithosphere of India shown in figure tomography, using the kinematic reconstruction of Tibet and the Himalaya of reference [9] and India–Asia convergence following reference [20].

The first detailed seismological section that detected horizontally underthrust lithosphere revealed that the Indian continent protrudes ∼400 km north of the southern Himalayan front [12]. Since then, multiple seismic tomography models have reproduced this finding but showed that the shape of the northern Indian margin is irregular, protruding ∼800 km northward at the longitude of the eastern Himalayan syntaxis, abruptly stepping southward to ∼400 km to the north of Bhutan and then increasing to ∼700 km again towards the longitude of the western syntaxis (Fig. 2) [7–11]. An onset of horizontal underthrusting can be calculated when assuming that the body of lithosphere below Tibet is a rigid part of the Indian plate, reconstructing India–Asia convergence, and correcting for Tibetan shortening. This predicts that the onset of horizontal underthrusting started around the Himalayan syntaxes ∼28 Ma and becomes gradually younger to ∼15 Ma at the longitude of Bhutan [9,14] (Fig. 3). Geological reconstructions of uplift, heating and resulting leucogranite intrusion in the Himalayan mountain range have been interpreted to reflect the lateral propagation of slab detachment a few Ma after the underthrusting of the modern Indian crust below Tibet, around 25 Ma for the eastern- and westernmost Himalaya, gradually younging towards 13 Ma in Bhutan [15]. This match suggests that the thick body of lithosphere below Tibet is indeed horizontally underthrust Indian lithosphere.

All Indian plate lithosphere that was consumed before Miocene horizontal underthrusting must thus have subducted into the mantle. There is broad consensus that the majority of this subducted lithosphere resides in the lower mantle below India, with a smaller and younger slab that was the last to detach, overturned in the mantle to the north of the main India slab (Fig. 2) [9,11,38,39,48]. An additional anomaly in the lower mantle below the equatorial Indian ocean has also long been interpreted as Neotethyan [28,38,39], but may instead be a relict of Mesozoic subduction between Tibetan blocks [14] (Fig. 2).

In summary, the paradox of the India–Asia collision is the following: there is no geological record of oceanic subduction that spanned the width of the orogen after initial collision ∼60 Ma and the system is therefore widely believed to have been fully continental since this time [11,22,23]; yet thousands of kilometers of Indian plate lithosphere was consumed without leaving an accretionary record and subducted deeply into the mantle, which are both typically associated with oceanic subduction and not previously demonstrated for continents [30]. Only the Indian plate lithosphere that arrived in the collision zone in the Early to Middle Miocene did not steeply subduct, but instead horizontally underthrusted below the upper plate.

### The controversy: scenarios for Indian plate paleogeography and subduction history

**Figure 4.**
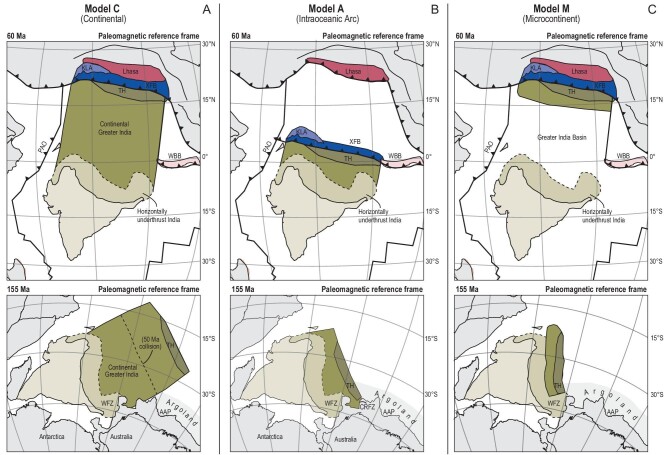
(A–C) Paleogeographic maps at the time of initial collision (∼60 Ma [17,18,43]) and in Gondwana fits at 155 Ma, corresponding to the timing of continental break-up in the Argo Abyssal Plain between northwest Australia and the conceptual Argoland continent [50] for three endmember models discussed in the text. Models are placed in the paleomagnetic reference frame of reference [100]. (A) Model C, with a fully continental Greater India [18,22,23,40]; (B) Model A, in which initial collision occurred with an intra-oceanic subduction zone around the equator. The size of continental Greater India is here constructed with a 40-Ma closure age of the remaining oceanic lithosphere [6,24–27] (Model C) in which a 60-Ma collision occurs between a microcontinent that broke off Northern India in the Cretaceous, opening a Greater India Basin in its wake [9,28]. AAP, Argo Abyssal Plain; CRFZ, Cape Range Fracture Zone; KLA, Kohistan–Ladakh Arc; PAO, Pakistan Ophiolites; TH, Tibetan Himalaya; WBB, West Burma Block; WFZ, Wallaby Fracture Zone; XFB, Xigaze Forearc Basin.

The above paradox has led to paleogeographic reconstructions for post-collisional Greater India that fall into three classes (Fig. 4). The first and most commonly portrayed scenario (Model C, for Continental) assumes that all post-collisional convergence consumed continental lithosphere [18,22,23,40]. This scenario provides a straightforward explanation for the absence of accretion of Ocean Plate Stratigraphy (OPS [49]) after 60 Ma in the Himalayan orogen but requires thousands of kilometers of continental subduction, and this subduction must have been accommodated along a continental subduction thrust, somewhere in the Himalaya [23]. The width of continental Greater India portrayed on published paleogeographic maps differs as a function of collision age, plate circuit, and assumed Tibetan shortening, but predicts Gondwana reconstructions in which Greater India was conjugate to the entire western Indian margin [23] up to or beyond the Argo Abyssal Plain (Fig. 4). This Argo Abyssal Plain is of importance because it recorded Jurassic continental break-up whereby the conceptual ‘Argoland’ continent whose remains now make up much of Indonesia and west Burma broke off Australia ∼155 Ma, well before the separation of India from Australia ∼130 Ma [50]. The Argo Abyssal Plane was thus conjugate to a different continent and plate than India. Based on marine magnetic anomalies and continental extension reconstructions of the west Australian margin, Gibbons *et al.* [50] suggested that Argoland must have continued as far south of the Wallaby Fracture Zone. Model C thus requires that their interpretation is incorrect.

The second scenario (Model A, for Arc) points out that between the Himalaya and continental southern Eurasia, there are ophiolites and intra-oceanic arc rocks, and invokes that the 60-Ma collision recorded the arrival of the north-Indian continental margin in an intra-oceanic subduction zone, followed by obduction of ophiolites and arc rocks onto the continental margin [6,16,24–27]. Following this collision, oceanic lithosphere remained between the initial collision zone and Eurasia, which was consumed until the arrival of the obducted Indian continental margin at the Tibetan trench. Because there is no accretionary record of post-60-Ma oceanic subduction, the age of this arrival is based on interpretations of changes in magmatism in Tibet, or a (contested) youngest age of marine sedimentation in the Himalaya, at 40 ± 5 Ma [6,25,27]. To explain how Tibet-derived sediments arrived at the north-Indian margin ∼60 Ma, a recent modification of this model suggested that the north Himalayan ophiolites originated at the south Tibetan margin in the Early Cretaceous but migrated southward, together with overlying Tibet-derived sediments, due to the opening of a back-arc basin [6]. The intra-oceanic arc scenario thus predicts that part of the post-collisional subduction history consumed oceanic lithosphere that must have subducted along a trench between the Himalayan ophiolites and the south Tibetan margin. Additionally, the assumed collision age of 40 ± 5 Ma of the obducted Indian margin and Tibet would still require large amounts (≤1000 km at the longitude of Bhutan) of continental subduction prior to horizontal underthrusting (Fig. 4). The reconstructed width of continental Greater India depends on the assumed collision age with Tibet but would bring the north Greater Indian margin adjacent to most of the west Australian margin up to the Cape Range Fracture Zone, thus also challenging Gibbons *et al.*’s [50] Argoland interpretation (Fig. 4).

The third scenario (Model M, for Microcontinent) invokes that the 60-Ma collision in the north Himalaya involves a Tibetan–Himalayan microcontinent that rifted and drifted away from Greater India in Cretaceous times, opening a conceptual Greater India Basin (GIB) ocean in its wake [28]. Assuming that the horizontally underthrust portion of India below Tibet represents the southern paleo-passive margin of this basin leads to a reconstruction whereby Greater India in Gondwana times did not extend beyond the Wallaby Fracture Zone of the southwest Australian margin [9], far south of the Argo Abyssal Plain, but consistent with west Australian margin reconstructions that interpreted that Jurassic break-up of Argoland to continue to the Wallaby Fracture Zone [50]. This model thus invokes that continental subduction was restricted to only the lower-crustal and mantle underpinnings of the Tibetan–Himalayan microcontinent. However, this model also requires that an oceanic basin was consumed along a subduction thrust within the Himalayan mountain range without leaving a modern geological record anywhere in the Himalaya. Finally, this scenario does not require, but also does not exclude, the intra-oceanic arc scenario of Model A—this would merely change the width of the GIB.

Each of these scenarios explains some first-order observations from the Greater Indian paradox and satisfies some long-held paradigms in subduction behavior or orogenesis, but challenges others. And each of these models has been defended as well as contested based on paleomagnetic, structural geological, stratigraphic and seismic tomographic data. Below is a brief review of the geological architecture of the Himalaya and Tibet that is relevant to identify future research targets to advance the discussion and to identify the main geological and geodynamic phenomena that occurred in the time window of horizontal Indian underthrusting.

### The constraints: architecture and evolution of the Tibetan–Himalayan orogen

**Figure 5.**
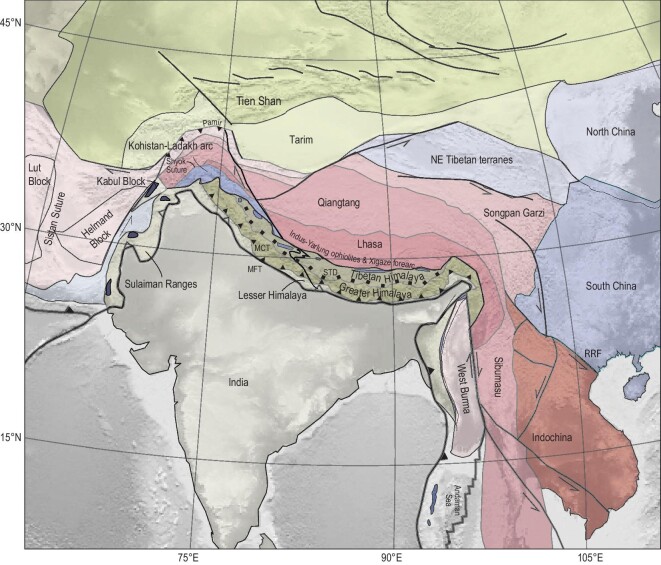
Tectonic map of the India–Asia collision zone, modified after reference [9]. MCT, Main Central Thrust; MFT, Main Frontal Thrust; RRF, Red River Fault; STD, South Tibetan Detachment.

Elements of the Himalayan and Tibetan orogen that play a key role in the interpretations of its tectonic history since 60 Ma are: (i) the accretionary fold-thrust belt of the Himalaya that was offscraped from now-underthrust/subducted Indian plate lithosphere; (ii) a belt of overlying ophiolites, and in the west of the collision zone, Cretaceous–Eocene intra-oceanic arc rocks that represent the upper plate of an overriding oceanic lithosphere above a subduction zone; and (iii) continental crust of the Tibetan Plateau that consists of pre-Cenozoic accreted terranes and intervening sutures, intruded by a Mesozoic–Cenozoic magmatic arc that also shows it was in an upper-plate position above a subduction zone (Fig. 5). These constraints and how they play a role in the three scenarios for Indian paleogeography are summarized below.

#### Himalaya

The accretionary fold-thrust belt of the Himalaya consists continent-derived nappes that underlie ocean-derived accreted units. These accreted rock units play a key role in reconstructing subducted plate paleogeography. Conceptually, accreted rock units fall into two broad types: ocean-derived units that consist of Ocean Plate Stratigraphy (OPS) comprising pillow lavas (MORB, OIB, IAT), pelagic oceanic sediments, and foreland basin clastics [49]. Continent-derived units consist of Continental Plate Stratigraphy (CPS) that in its simplest form comprises slivers of a basement from an earlier orogenic cycle, an unconformable cover of syn-rift clastic sediments and volcanics, shallow-marine to deep-marine platform to pelagic passive margin carbonates and occasional clastic series, and foreland basin clastics, although a more complex stratigraphic architecture may form due to climatic or relative sea-level variation or a more complex rifting history of the continental margin [30]. Key for analysing the collision and accretion history are the foreland basin clastics: these not only date the arrival of the accreted units at a trench, but also allow fingerprinting the nature of the overriding plate through sediment provenance analysis. The moment of accretion of thrust slices is bracketed between the youngest flysch deposits giving a maximum age and, if burial was deep enough, the age of metamorphism (in subduction setting normally of HP–LT type, except during subduction infancy, when HT–HP metamorphic soles may form [51]) of the accreted units, which gives a minimum age [30]. Finally, in fold-thrust belts with continuous foreland-propagating thrusting in which almost all subducted lithosphere left its upper crust in the orogen, the youngest age of foreland basin clastics in the higher nappe tends to be similar to the oldest age of foreland basin clastics in the next-lower nappe (as for instance in the Apennines and Hellenides of the Mediterranean region [52]). Conversely, extended periods of non-accretion and wholesale subduction, or subduction erosion removing previously accreted rocks, are revealed by age gaps between foreland basin clastics in adjacent nappes (e.g. in the Japan accretionary prism [49]).

The Himalayan fold-thrust belt is commonly divided into four main units, three of which follow the logic outlined above. The highest unit, located below the Indus–Yarlung ophiolites, is a mélange that consists of deformed and in places metamorphosed OPS. These include pillow basalts, cherts that are no older than Triassic in age reflecting the age of the opening of the Neotethys ocean [53] and foreland basin clastics in which the youngest recognized ages are ∼80 Ma [54]. The first-accreted units are dismembered metamorphic sole rocks with ∼130 Ma ^40^Ar/^39^Ar cooling ages that provide a minimum age for subduction initiation [55]. HP–LT metamorphic OPS units found in the mélange below the ophiolites interpreted to have formed during oceanic subduction have ages of 100–80 Ma [43].

This OPS-derived mélange overlies the Tibetan–Himalayan nappe. This nappe consists of upper Proterozoic to Paleozoic basement, upper Paleozoic syn-rift clastics and volcanics, a carbonate-dominated passive margin sequence that continues into the Cenozoic [56] and Paleocene to lower Eocene foreland basin clastics whose age estimates range from ∼61 to 54 Ma [17,18,57]. Metamorphic ages of (U)HP metamorphic, deeply underthrust equivalents of the Tibetan–Himalayan, reveal ages suggesting that burial was underway by 57 Ma [43] and continued until at least ∼47 Ma [58]. These records provide evidence that continental lithosphere on the Indian plate arrived in a subduction zone by ∼60 Ma or shortly thereafter.

**Figure 6.**
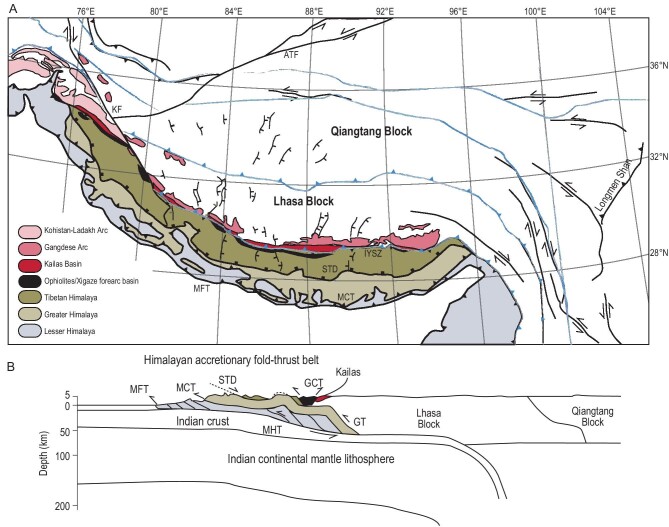
(A) Tectonic map of the Himalaya and Tibet, simplified after references [55,85,86]. (B) Schematic cross section through the Himalaya and southern Tibet, modified from reference [6]. ATF, Altyn Tagh Fault; GCT, Great Counter Thrust; GT, Gangdese Thrust; IYSZ, Indus–Yarlung Suture Zone; KF, Karakoram Fault; MCT, Main Central Thrust; MFT, Main Frontal Thrust; MHT, Main Himalayan Thrust; STD, South Tibetan Detachment.

The Tibetan–Himalayan nappes overlie crystalline rocks of the Greater Himalaya. These Greater Himalayan rocks are atypical for accretionary fold-thrust belts in their metamorphic grade as well as in their stratigraphy. They consist of upper Proterozoic sedimentary rocks intruded by lower Paleozoic granitoids, which were both metamorphosed in Cenozoic times under high-grade metamorphic conditions, up to partial melting, and intruded by leucogranites [6,59–61]. In the structurally higher portions of the Greater Himalayan rocks, prograde metamorphism from ∼50 Ma onward has been demonstrated, showing that they have been part of the orogen since at least Early Eocene time [59,62]. The top of the Greater Himalayan sequence thus likely represent the original stratigraphic underpinnings of the Tibetan–Himalayan sequences [15]. Ages recording peak metamorphism become younger from top to bottom across thrusts within the Greater Himalaya, spanning ages from the Eocene to the Early Miocene [63], which may suggest step-wise accretion of nappes from the subducting Indian plate [15]. However, there is no record of a Mesozoic passive margin stratigraphy or of Cenozoic foreland basin clastics in the Greater Himalayan rocks [6,61]. Because accretion is a top-down process and it is not possible to accrete the deeper part of the stratigraphy without accreting the shallower part, it is thus unlikely that the Greater Himalayan sequence contains separate, far-traveled CPS-bearing nappes that were derived from lithosphere paleogeographically to the south of the Tethyan Himalaya [30]. Instead, the downstepping and thrusts likely reflects slow, post-accretion upper-plate shortening and burial as part of the thickening Tibetan Plateau. The Greater Himalayan sequence is separated from the overlying Tethyan Himalayan sequence by a ductile shear zone that is known as the South Tibetan Detachment (STD), which has been active in latest Oligocene to Middle Miocene time [59] and was interpreted as a normal fault accommodating exhumation and channel flow [60] (Fig. 6) or as an out-of-sequence thrust that displaced the Tethyan Himalayan top relative to its Greater Himalayan underpinnings [15].

The base of the Greater Himalaya is the Main Central Thrust (MCT)—a ductile shear zone that is the youngest thrust of the Greater Himalayan sequence. It has a downward decreasing metamorphic grade, signaling syn-exhumation activity, that reveals ages of latest Oligocene to Middle Miocene (∼26–13 Ma) activity coeval with the STD [59,60]. The coeval activity of the MCT and STD is commonly (but not exclusively [15]) interpreted to reflect extrusion of a mid-crustal part of the orogen [64] that slowly heated up following burial since the Eocene [59]. During Miocene exhumation, the Greater Himalayan crystalline rocks were emplaced onto the Lesser Himalayan sequence that contain Lower Miocene foreland basin clastics (see below) and were accreted to the orogen since the Middle Miocene.

The Lesser Himalaya consists of a Proterozoic to Paleozoic, low-grade metasedimentary, and discontinuous Cretaceous-to-Paleocene clastic sedimentary rocks, in places overlain by Eocene and Miocene foreland basin clastics [57]. Upper Cretaceous-to-Eocene clastic sedimentary rocks become more prominent towards the west, in Pakistan, where Eocene and younger foreland basin clastics are also found on the undeformed Indian continent [65,66]. The provenance of Upper Cretaceous and Eocene foreland basin clastics in the Lesser Himalayas and on the northwest Indian continent reveal erosion of Indian margin rocks and ophiolites that signal Eocene or older obduction, and is commonly interpreted to reflect collision recorded in the Tethyan Himalaya to the north [33,57,65,66]. However, the western margin of India was also the locus of orogenesis due to ophiolite emplacement in a Late Cretaceous and an Eocene phase, but this obduction was governed by convergence between the Indian and Arabian plates and the collision of the Kabul microcontinent with west India [67]. So far, the sediment provenance studies have not identified whether the west and north-Indian margin have distinctly different signatures presenting an unresolved challenge in interpreting sediment provenance [9]. Duplexing of the Lesser Himalayan rocks occurred in the last ∼15–13 Ma and accounted for hundreds of kilometers of shortening that is similar to contemporaneous Indian plate consumption [42,68].

**Figure 7.**
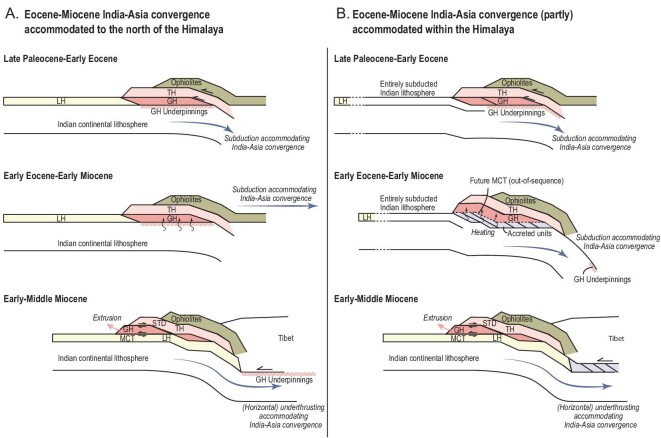
Conceptual evolution of Himalayan architecture if (A) all Eocene–Early Miocene India–Asia convergence is accommodated to the north of the Himalaya. In this case, the MCT can have formed when the Greater Himalayan rocks decoupled from their original Indian lower-crustal and lithospheric underpinnings or (B) all or part of the Eocene–Early Miocene India–Asia convergence is accommodated within the Himalaya. In this case, the MCT is an out-of-sequence thrust that formed within the Early Miocene Himalayan fold-thrust belt and Eocene–Miocene units that may have accreted below the Greater Himalaya have been removed by subduction erosion.

The structure of the Himalaya summarized above show an overall foreland-propagating fold-thrust belt, but with a clear omission of accretion between the Eocene (Tibetan and Greater Himalaya) and Miocene (Lesser Himalaya). There are two endmember interpretations of this hiatus in accretionary record. Before their Miocene emplacement onto the Lesser Himalaya, the rocks exposed in the Greater Himalaya must have been overlying rocks that have now been transported farther below the orogen and the nature of these rocks is unknown. On the one hand, these rocks may have been the original underlying Indian basement [22,68] (Fig. 7). In that case, there has been no net convergence between the Greater and Lesser Himalaya between Eocene burial of the former and Miocene burial of the latter. The Eocene–Miocene India–Asia plate boundary must then have been located north of the Himalaya. Of the three models for Indian paleogeography (Fig. 4), only Model A (intra-oceanic arc) could allow for this scenario: in that case, Early Eocene burial of the Greater Himalaya follows upon obduction and activation of the MCT would reflect final collision of the obducted margin with Tibet—but this would require a diachronous Miocene collision age, instead of the proposed collision ages of 40 ± 5 Ma. All other scenarios require that a subduction plate boundary (intra-continental, or ocean-below continent) existed within the Himalaya. In that case, the Greater Himalayan sequence must have decoupled from its Indian basement sometime after its Early Eocene arrival in the orogen and subsequently formed part of a slowly thickening and heating orogen. This may be consistent with the evidence for downstepping thrusting and progressively younger metamorphic ages from top to bottom throughout the Paleogene [15,63]. The activation of the Miocene MCT was then the youngest of these downstepping thrusts and decoupled the modern Greater Himalayan in the hanging wall from its pre-Miocene underpinnings that traveled deeper below the orogen, followed by accretion of the Lesser Himalayan foreland basin and deeper stratigraphic units. Such a scenario is typically implied in numerical simulations of Himalayan extrusion and channel flow [69] and interprets the MCT, and the older intra-Greater Himalayan thrusts, as out-of-sequence thrusts in a shortening and thickening upper plate (Fig. 7). Importantly any Eo-Oligocene accretionary record and associated thrusts that formed below the Greater Himalayan sequence were then removed from the orogen, i.e. essentially through subduction erosion [70], upon activation of the MCT (Fig. 7). In Models C and A, this removed part of the orogen that consisted of accreted CPS, in Model M (microcontinent), may also have included OPS.

#### Indus–Yarlung ophiolites and Kohistan–Ladakh arc

Overlying the accretionary orogen of the Himalaya is a series of ophiolites concentrated in a narrow belt along the northern Himalaya [6] (Figs 5 and 6). These ‘Indus–Yarlung’ ophiolites are predominantly Early Cretaceous in age (∼130–120 Ma), during which time they formed by extension in the forearc above a (presumably incipient) subduction zone [6,55]. In some places also older, Jurassic oceanic crust is found in ophiolites, which may reflect the ocean floor trapped above the subduction zone, in which the Cretaceous ophiolites formed [6]. In addition, to the northwest of the Himalaya, a long-lived intra-oceanic arc sequence (150–50 Ma) that is located between the ophiolites and the continental units of southern Eurasia is known as the Kohistan–Ladakh arc [71]. These sequences showed that the accretion of the Himalayan rocks occurred below a forearc that consisted of oceanic lithosphere, which plays a central role in the controversy about Greater Indian paleogeography.

The Kohistan–Ladakh arc is overlain by a Cretaceous-to-Eocene sedimentary sequence and is separated from Tibetan continental rocks by the Shyok Suture (Fig. 5). Convergence across this suture zone has been proposed to be either significant and continuing to Eocene time [26,27] or minor and pre-dating the Late Cretaceous [32], but in any case testifies to the existence of a paleo-subduction zone between the Kohistan–Ladakh arc and Eurasia. The Indus–Yarlung ophiolites are overlain by sediments of the Xigaze forearc basin that form a major syncline with 4–5 km of sediments along 550 km of the suture zone [72,73]. The oldest sediments are ∼130 Ma old and unconformably overlie exhumed oceanic core complexes of the ophiolites and elsewhere interfinger with the ophiolites’ pelagic sedimentary cover [74], and the youngest part of the continuous section is ∼50 Ma [72,73]. Low-temperature thermochronology revealed that the succession may have been almost twice as thick and suggested that sedimentation and burial may have continued until ∼35 Ma [72]. The Xigaze forearc has been shortened along the north-dipping Gangdese Thrust, which brought Tibetan rocks over the forearc between ∼27 and 23 Ma [75], and the Great Counter Thrust that backthrusted the Xigaze forearc over the south Tibetan margin between ∼25 and 17 Ma [6] (Fig. 6). Sediment provenance studies of the Xigaze forearc sequence typically depict southern Tibet and its overlying magmatic arc as source [72–74], although others prefer an intra-oceanic arc derivation [25,26] and there is no known accretionary record of OPS or mélange along the strike of the northern margin of the Xigaze forearc basin that may reflect the location of a post-60-Ma paleo-subduction zone.

The Indus–Yarlung ophiolites have been interpreted as the forearc of the Eurasian plate, whereby they formed by (hyper)-extension of the Tibetan continental lithosphere, occasionally trapping ocean floor that existed before subduction initiation next to the south Tibetan passive margin [76,77]. In this case, the Kohistan–Ladakh arc forms an along-strike, intra-oceanic continuation of a contemporaneous arc in Tibet (the Gangdese arc, Fig. 6) and the Shyok Suture accommodated only minor convergence that eastwards was accommodated within the Tibetan Plateau [9,32]. This scenario is required by Model C (fully continental Greater India) and preferred by Model M (microcontinent). On the other hand, Model A predicts that the Kohistan–Ladakh arc and Indus–Yarlung ophiolites formed at (or migrated to [6]) equatorial latitudes, far south of the south Tibetan margin, at a separate subduction zone [25–27]. This model predicts major convergence across the Shyok Suture, but requires that a long-lived subduction zone is hidden between the Xigaze Basin and the adjacent south Tibetan margin.

#### Tibetan Plateau

The Tibetan Plateau consists of a series of Gondwana-derived continental fragments and intervening suture zones that amalgamated in Mesozoic time [6,78]. The southernmost of these fragments is the Lhasa Block that accreted to the Tibetan Plateau in Early Cretaceous time [6,78], around the same time as the formation of the south Tibetan ophiolites above a nascent subduction zone to the south of Lhasa [55]. Shortening of the Tibetan upper plate above this subduction zone started in Late Cretaceous time [79–81] and amounted perhaps 400 km before initial collision [41] in addition to the 1000–1200 km of post-60-Ma shortening [9,41]. Detailed stratigraphic records reveal that shortening in the plateau may have been pulsed but there is no evidence of a shortening pulse associated with initial collision ∼60 Ma; the recorded pulses may rather reflect changes in Indian Plate subduction rate [20,80]. In Eocene–Oligocene time, shortening was concentrated in the central Tibetan Plateau. Sometime in Late Eocene or Oligocene time (∼30 ± 7 Ma), Tibetan shortening started to affect the southern margin of the rigid Tarim block to the north of the modern plateau. To the west of this block, Eurasian lithosphere started to subduct southward, whereas to the southeast of Tarim, Tibetan crust started to move northeastward along the Altyn Tagh fault [82]. In Late Oligocene time, ∼25 Ma, shortening propagated beyond the Tarim block into the Tien Shan, intensifying at ∼13–10 Ma [83]. Throughout this history, also northeastern Tibet underwent outward growth by foreland-propagating thrusting [6,84].

Paradoxically, even though the Tibetan Plateau and Tien Shan underwent ongoing shortening in Oligocene-to-Early-Miocene time, south-central Tibet experienced dynamic subsidence, or even extension. On the southern margin of the Lhasa Block, close to the suture zone, formed the 1300-km-long Kailas Basin, which forms a southward thickening wedge of >3 km of sediments. The basin's architecture and sedimentology suggest that it formed in the hanging wall of a north-dipping normal fault, even though the fault itself is not exposed, perhaps cut out by the Great Counter Thrust [85,86] (Fig. 6). The stratigraphy in any section of the basin accumulated within only 2–3 Ma, but the timing of basin formation propagates diachronously along-strike, between 26 and 24 Ma in the west, and becoming as young as 18 Ma in the east [86].

Upper-plate deformation in Tibet also involved lateral extrusion [40]. In the east of the plateau, crust was extruded eastwards already in the Eocene, first accommodated by rotations and thickening in northwest Indochina and later, sometime between ∼30 and 15 Ma, also by motion of entire Indochina along the Red River Fault [87] (Fig. 5). In western Tibet, a similar process may have played a role, although the lack of detailed knowledge of the geology of Afghanistan limits constraints [24]. A recent reconstruction of Central Iran [88] pointed out major Late-Cretaceous-to-Eocene mobility and east–west (E–W) convergence across the east Iranian Sistan suture requires that continental fragments of Afghanistan may have undergone major westward displacement (Fig. 5). Restoring such displacement would bring the Afghanistan fragments north of the Kohistan–Ladakh arc and is thus relevant in interpreting its paleolatitudinal history in terms of Greater Indian paleogeography, but awaits future detailed constraints.

Around 15–10 Ma, a prominent change in deformation of the Tibetan Plateau occurred, which most famously marks the onset of regional E–W extension in the plateau interior [89,90] (Fig. 6). Towards the west, this extension is bounded by the Karakoram Fault that accommodated ongoing convergence in the Pamir region [41] (Figs 5 and 6) and to the east, it is accommodated by E–W shortening in the Longmenshan range, and by a deflection of motion towards the Yunnan region in the southeast, accommodated along major strike-slip faults [2,90]. This motion is prominent today as reflected by GPS measurements. Eastward surface motion components increase from near-zero at the Karakoram Fault eastward to a maximum of ∼2 cm/yr on the central plateau [91]. Eastward motion components then decrease farther to the east due to an increasing southward velocity component in eastern Tibet, as well as E–W shortening in the Longmenshan [90,91]. The extension of the plateau interior and the motion of crust towards the southeast is widely interpreted as driven by excess gravitational potential energy resulting from plateau uplift [2,45], facilitated by a partially molten middle crust [92]. The trigger of extension is thought to reflect Middle Miocene uplift of Tibet due to lithospheric delamination [2,45,90] or due to horizontal Indian continental underthrusting [13].

Finally, the Lhasa terrane contains the prominent Gangdese batholith that represents a long-lived volcanic arc [6] (Fig. 6). Arc magmatism in the Lhasa terrane related to Neotethys closure has been active since at least Early Cretaceous time and perhaps longer [6]. Magmatism of the Gangdese arc since Early Cretaceous time contained flare-ups and periods of reduced activity, but was mostly active until ∼45–40 Ma, after which there was a lull until 25 Ma [3,6]. During this lull, potassic and ultrapotassic magmatism was active in the Qiangtang terrane, hundreds of kilometers to the north of the Gangdese batholith, after which magmatism resumed in the Lhasa terrane, ultrapotassic or shoshonitic/adakitic in composition [3,6], associated with economic porphyry copper deposits [4]. Since 20 Ma, such magmatism also resumed in the Qiangtang and adjacent Songpan Garzi zones of the Tibetan Plateau [3]. Interestingly, this Miocene magmatism in the Lhasa terrane migrated eastward, 25–20 Ma in western Tibet but 15–10 Ma in the east, towards the longitude of Bhutan [5]. The chemistry of these magmatic rocks is interpreted to be mostly derived from a previously subduction-enriched asthenospheric source that became stirred by the underthrusting continental Indian lithosphere [3–5].

## DISCUSSION

### Opportunities 1: natural laboratory of converging unsubductable lithospheres

The kinematic reconstruction constraining horizontal continental underthrusting of the Indian continent below Tibet identifies (only) the Miocene and younger Tibetan–Himalayan geological history as a natural laboratory for the convergence of unsubductable lithospheres. While an extensive analysis of the dynamics of this system is beyond the scope of this paper, several first-order temporal and spatial relationships between horizontal underthrusting and geological evolution are clear and may be used as a basis for discerning between existing hypotheses or developing new ones.

Most importantly, the irregular shape of the seismically imaged northern Indian continental margin shows that initial horizontal underthrusting must have been diachronous: the coinciding age estimates from the kinematic restoration of this margin [14] (Fig. 3) and geological estimates of the youngest phase of slab break-off from the Himalaya [15] of ∼25 Ma at the Himalayan syntaxes, decreasing to ∼13 Ma in at the longitude of Bhutan, may provide means to discern between the effects of horizontal underthrusting and unrelated events. For instance, the reinitiation of magmatism between 25 and 8 Ma in the Lhasa terrane follows the same age progression, lending independent support to the interpretation that magmatism resulted from incipient Indian continental lithosphere plowing through and stirring a previously subduction-enriched asthenosphere [3–5,93]. On the other hand, Miocene magmatism farther north in the Tibetan Plateau that started ∼20 Ma is located far away from the horizontally underthrusting northern Indian continental margin and does not show a lateral age progression, making a direct link unlikely.

**Figure 8.**
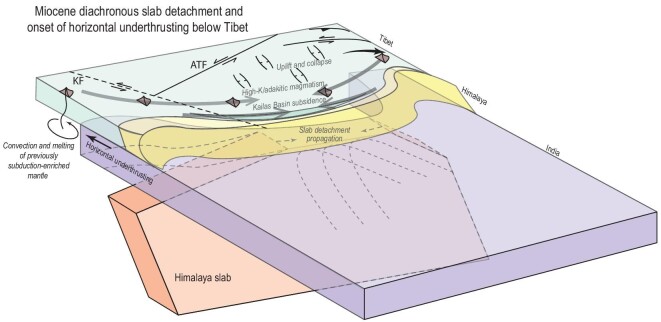
Cartoon illustrating geometrical relationships between diachronous slab detachment and onset of horizontal Indian continental lithospheric underthrusting below Tibet between 25 and 13 Ma, and geological expressions in the Tibetan Plateau.

The formation and deposition of the Kailas Basin follow the same diachronous trend but precede the reconstructed slab break-off by a few Ma [86]. The recognition of diachronous initial horizontal underthrusting allows explaining this trend, as well as the apparent paradox of north–south extension in the Kailas Basin of southern Tibet [85,86] and the coeval ongoing upper-plate shortening in the Pamir, along the Altyn Tagh fault, and in northeastern Tibet [82,84]. The subsidence of the Kailas Basin is well explained as the result of negative dynamic topography, or even upper-plate extension, caused by the Himalayan slab retreating and steepening relative to the upper plate, which was previously interpreted to reflect slab rollback [86,94]. Slab rollback, however, would lead to slabs horizontally draping the upper mantle–lower mantle transition zone, whereas the Himalaya slab is overturned northward, which requires slab advance during subduction, prior to detachment [14] (Fig. 8). But slab advance resisting upper-plate retreat would generate the same relative slab–upper-plate motion as envisaged before for Kailas [86,94]. This resistance only occurs where the slab is still attached, explaining diachroneity in Kailas Basin formation and its subsequent uplift. But where slab detachment had already occurred, i.e. at the longitude of the Himalayan syntaxes in the Pamir and eastern Tibet, horizontal Indian underthrusting may already have caused enhanced friction to drive the apparently paradoxical simultaneous upper-plate shortening and extension (Fig. 8).

The reconstructed horizontal Indian underthrusting also sheds light on the long-standing debate on the trigger of E–W extension in Tibet. There is widespread consensus that this extension reflects the gravitational collapse of the Tibetan Plateau [2,13,45,90], alongside orogen-parallel extension in the Himalaya due to oroclinal bending [15]. As a final trigger to drive collapse, lithosphere delamination of south-central Tibet [2,45,90] or enhanced plateau uplift due to horizontal Indian underthrusting [13] have been suggested. Horizontally underthrusting Indian continental lithosphere directly underlies Tibetan crust and Tibetan lithospheric mantle must thus have delaminated prior to the 25-Ma onset of horizontal underthrusting in western and eastern Tibet. In addition, not only the source area below the Tibetan Plateau, but also the ‘sink’ of Middle Miocene and younger crustal motion in the Yunnan region has undergone lithospheric delamination [47]. This suggests that the onset of E–W extension 15–10 Ma was likely not triggered by delamination. More likely, collapse was driven by the final onset of horizontal underthrusting below the entire plateau following final slab break-off [13]. If horizontal underthrusting indeed caused uplift, the easternmost part of the Indian continental promontory north of the eastern syntaxis may have first formed a barrier against plateau collapse, which was only overcome after the entire Tibetan Plateau became horizontally underthrust by India since Middle Miocene time.

Also Middle Miocene changes in the Himalaya may be studied in the context of the transition from subduction to horizontal underthrusting. Webb *et al.* [15] already interpreted syntaxis formation and Himalayan oroclinal bending as a result of the change to horizontal underthrusting. Also the transition from extrusion of the Greater Himalayan crystalline rocks along the STD and MCT to duplexing of the Lesser Himalayan nappes appears to coincide with the transition to horizontal underthrusting, but future analyses may test whether there was diachroneity in these processes. The coincidence of intraplate deformation events, e.g. in the Tien Shan with the onset of horizontal underthrusting in western Tibet ∼25 Ma and along the entire Tibetan margin ∼13 Ma, may suggest a causal relationship linking convergence between unsubductable lithosphere to intraplate deformation. On the other hand, the shortening in the Tien Shan may also be a natural northward progression of intraplate deformation that had long been ongoing in the Tibetan Plateau. Future numerical experiments may test such dynamic hypotheses built on the Miocene Tibetan–Himalayan natural laboratory for the convergence of unsubductable lithosphere.

### Opportunities 2: improving methodology to unlock the post-collisional subduction laboratory

The ongoing controversy of Greater Indian paleogeography currently hampers using the interval between initial collision ∼60 Ma and the horizontal Indian underthrusting 25–13 Ma as a conclusive natural laboratory for post-collisional subduction. Regardless which of the Models C, A or M will turn out to be correct, if any, this natural laboratory holds great promise. Models C and A so far offer no explanation for why there was a transition from subduction to horizontal underthrusting, or what caused the diachroneity of that transition, but if these scenarios are correct, that explanation must provide a unique constraint on the subductability of continental lithosphere. Moreover, Models C and A predict that continental subduction is also possible without preservation of upper-crustal units, or with large-scale subsequent removal of accreted continental crust through subduction erosion. If these models are correct, it is thus possible that paleogeographic reconstructions strongly underestimate the paleogeographic area occupied by continental lithosphere. In fact, if large portions of continental lithosphere can subduct without leaving a geological record, accreted geological records such as in the Tibetan Himalaya cannot provide conclusive constraints on initial collision, but only give a minimum age [30]. Finally, Model C (since 60 Ma) and Model A (since 40 ± 5 Ma) would provide the opportunity to calibrate magmatic responses to continental subduction.

The subduction history of Model M is on a par with the geodynamic and paleogeographic paradigm that continental lithosphere generally does not subduct and that if it does, its upper crust will accrete in orogenic belts [29,52]. The short-lived, Late-Paleocene-to-Early-Eocene phase of microcontinental lower-crust and mantle-lithosphere subduction combined with upper-crustal accretion is an example of the latter. In Model M, upper-crustal nappes of all subducted or horizontally underthrust continental lithosphere still remain in the Himalayan orogen [9]. The transition from subduction to horizontal underthrusting in Model M is simply caused by the change from oceanic to continental subduction. But Model M invokes that the anomalous magmatic history of Tibet between 45 Ma and the 25-Ma onset of horizontal underthrusting occurred during oceanic (perhaps flat-slab [9,86]) subduction and would thus allow calibrating possible magmatic arc expressions of anomalous oceanic subduction.

The three models provide strongly different boundary conditions and have far-reaching consequences for the analysis of the dynamic drivers of upper and intraplate deformation, the causes of rapid plate motion changes of India or the causes and paleogeographic context of terrestrial biota exchange and radiation. It is therefore important to attempt at breaking through the impasse in Greater Indian paleogeography reconstruction.

The only quantitative constraint on paleogeographic position comes from paleomagnetic data providing paleolatitudinal control. Paleomagnetic analyses on rocks derived from Greater India such as the Tibetan–Himalayan sequence, of ophiolites and intra-oceanic arcs and their cover, and of the Lhasa terrane of southern Tibet in principle allow discerning between Models C, A and M. But each of these models has been defended and challenged based on paleomagnetic data [26,28,34–37]. So are paleomagnetic data inconclusive? Rowley [34] recently pointed out that the widely used method to compare paleomagnetic study means (‘paleopoles’) with apparent polar wander paths that provide the global reference against which these data are compared and that are based on averages of study means is indeed barely conclusive. The paleopoles underlying apparent polar wander paths (APWPs) are scattered by ∼20° around the mean and Rowley [34] argued that individual paleopoles cannot constrain paleolatitude at a higher resolution. Vaes *et al.* [95], however, recently analysed the source of this scatter and showed that alongside common paleomagnetic artifacts such as undersampling of paleosecular variation and inclination shallowing in sediments, scatter is predominantly caused by the degree to which paleosecular variation is averaged: scatter is a function of the number of paleomagnetic datapoints used to determine a paleopole. And because this number is arbitrary, the statistical properties of APWPs calculated from paleopoles are arbitrary. Vaes *et al.* [95] provided a way forward in which paleopoles are compared to a reference curve that is also calculated from paleomagnetic spot readings rather than paleopoles, and developed a comparison metric that demonstrates a paleolatitudinal difference or vertical-axis rotation with 95% confidence. This would provide a means to compare data sets of unequal magnitude and propagate uncertainties, and may provide a more conclusive, quantitative and robust paleomagnetic analysis that may discern between the Greater Indian paleogeography models. Applying this analysis will likely decrease the scatter in paleomagnetic estimates of paleolatitude, provide more realistic error margins to discern relative motion between Himalayan units and India, and will demonstrate with a 95%, rather than ∼50%, certainty on whether a difference between a paleopole from the collision zone and India or Eurasia demonstrates tectonic motion or not.

Models C, A and M each invokes that a plate boundary must have existed south of the Tibetan Plateau between the Paleocene-to-Early-Eocene accretion of the Tibetan and Greater Himalayan units in the orogen and the accretion of the Miocene Lesser Himalayan units. If this plate boundary was located in the Himalaya during all or some of the period between 60 and 25/13 Ma, as currently required by all three scenarios, there may be no record due to out-of-sequence thrusting along the MCT removing the pre-Miocene underpinnings (Fig. 7). But this refocuses the attention on the process of extrusion and channel flow, this time not to explain the presence of the Greater Himalayan rocks in the orogen, but to explain the absence of its pre-Miocene underpinnings. In addition, Model A required that a subduction plate boundary was present between the Xigaze forearc and underlying ophiolites, and the Lhasa terrane after 60 Ma [6]. Detailed mapping, or identifying structures that could explain the lack of a record such as for the MCT (Fig. 7), may establish whether, when, and where such a subduction zone may have existed.

Also, sediment provenance studies have been used to argue for and against Models C, A and M. Part of this may underlie the qualitative nature of comparing e.g. detrital geochronology peaks between the sedimentary record of a sink and a suspected source area, and recently developed quantitative approaches that identify the likelihood of the contribution of a given source area to a sediment may advance the discussion [96]. In this analysis, the range of possible source areas for sediments, particularly for Eocene stratigraphic records in the northwestern Lesser Himalaya and the Pakistani foreland, should include not only the Himalaya–Kohistan–Ladakh–Tibetan orogen at the India–Asia plate boundary, but also the Sulaiman–Kabul Block orogen and associated ophiolites that formed independently at the India–Arabia plate boundary [67] (Fig. 4). In addition, provenance studies may benefit from broadening the time and space windows of the investigation. For instance, Triassic sandstones of the northeastern Tibetan Himalaya were interpreted to have a provenance of western Australia rather than northern Australia [97], but this conflicts with the interpretation that lower Eocene sediments in the Lesser Himalaya and on the Indian foreland include sediments derived from the north of the Shyok Suture [33]. Paleogeographic predictions like those for Models C, A and M show the paleogeographic implication farther back in time of interpretations for the Cenozoic and including these in the analysis may resolve apparent conflicting interpretations based on the same data types [33,97].

Seismic tomographic records of subducted slabs are useful in identifying regions of paleo-subduction [38,39], although global correlations suggest that the lower mantle hosts slabs of the last ∼250 Ma [48]. Analysis of mantle structure should hence be done in the context of Mesozoic and Cenozoic subduction history and uncertainties therein [14] (Fig. 2). Nonetheless, a recent seismological study of a slab below Kamchatka was able to identify thick crust in the order of 20 km in a lower-mantle slab [98]. Once a slab can be firmly tied to lithosphere that subducted after initial collision, such as the overturned Himalayan slab that straddles the transition zone [11,38], such seismological analyses may provide novel constraints on their composition and crustal nature.

In summary, on the one hand, the current controversy on Indian paleogeography stemming from the inability of geological and geophysical techniques to conclusively identify between vastly different paleogeographic scenarios stands in the way of using the India–Asia collision zone to calibrate the geological and dynamic responses to post-collisional subduction. On the other hand, this controversy provides the opportunity (and requires) to question and improve geological methodology to constrain paleogeography, including orogen structure, sediment provenance analysis, and paleomagnetism. Solving those issues has impact far beyond the analysis of the India–Asia collision history.

## CONCLUSIONS

Seismological images reveal that 400–800 km of Indian continental lithosphere is currently horizontally underthrust below Tibet. Using plate reconstructions that incorporate Tibetan shortening predicts that the onset of horizontal underthrusting started ∼25 Ma around the Himalayan syntaxes, gradually younging to 13 Ma at the longitude of Bhutan. This reconstruction coincides with independent estimates of diachronous slab break-off in the Himalaya and identifies the Miocene history of Tibet as a natural laboratory for the convergence of unsubductable lithospheres. This time period was marked by major changes in accretionary style in the Himalaya, including the extrusion of the Greater Himalayan crystalline rocks and the transition to Lesser Himalayan duplexing, but also by the onset of E–W extension and collapse of the Tibetan Plateau, and upper-plate shortening reaching as far north as the Tien Shan. Also, marked changes in magmatism in southern Tibet and associated economic mineralizations spatially and temporally correlate with the reconstructed inception horizontal underthrusting. These processes may provide key ingredients of the natural laboratory for convergence of unsubductable lithosphere. Importantly, lithospheric delamination of Tibet, often cited as a potential trigger for Miocene Tibetan uplift and collapse, must instead have occurred prior to horizontal Indian underthrusting, hence before the Miocene.

Between initial collision recorded in the Himalaya at 60 Ma and the onset of horizontal Indian underthrusting, thousands of kilometers of subduction consumed Indian plate lithosphere. Three endmember scenarios invoke that all or part of this lithosphere was continental, challenging geodynamic and paleogeographic reconstruction paradigms, or that most of this lithosphere was oceanic, challenging magmatic and orogenic architecture paradigms. But an impasse is reached because each of these reconstructions is argued for and against based on the same data types. There are opportunities for methodological advances in fields including paleomagnetism, sediment provenance analysis and seismology to overcome this impasse, unlocking the interval of Tibetan and Himalayan evolution of 60–25/13 Ma as a natural laboratory for typical geological responses for atypical post-collisional subduction or for atypical geological responses to typical oceanic subduction.
